# FTIR Spectroscopy of Vitreous Humor for Postmortem Interval Estimation: A Multivariate Regression Approach

**DOI:** 10.3390/ijms27083468

**Published:** 2026-04-13

**Authors:** Ioana Ruxandra Țurlea, George Cristian Curca, Maria Mernea, Alina Cristina Mătanie, Sergiu Fendrihan, Dan Florin Mihăilescu

**Affiliations:** 1 Department of Legal Medicine and Bioethics, “Carol Davila” University of Medicine and Pharmacy, 8 Eroii Sanitari Boulevard, 050474 Bucharest, Romania; ioana-ruxandra.turlea@umfcd.ro (I.R.Ț.); george.curca@umfcd.ro (G.C.C.); 2 ”Mina Minovici” National Institute of Legal Medicine, 9-11 Vitan-Bârzești Road, 077160 Bucharest, Romania; 3 Department of Anatomy, Animal Physiology and Biophysics, Faculty of Biology, University of Bucharest, 91–95 Splaiul Independenței, 050095 Bucharest, Romania; cristina.matanie@bio.unibuc.ro (A.C.M.); d.f.mihailescu@gmail.com (D.F.M.); 4 Non-Governmental Research Organization Biologic, 14 Schitului Str., 032044 Bucharest, Romania; ecologos23@yahoo.com

**Keywords:** FTIR spectroscopy, postmortem interval (PMI), human vitreous humor, multivariate regression, peak intensity analysis, forensic science

## Abstract

Estimation of the postmortem interval (PMI) remains a major challenge in forensic science. We used attenuated total reflection (ATR)–Fourier-transform infrared (FTIR) spectroscopy combined with chemometric modeling for PMI prediction using vitreous humor samples from 20 forensic cases with known PMI (24.8–97.6 h) and 10 with unknown PMI. The intensities of vibrational bands commonly associated with PMI were analyzed, and several peaks in the carbohydrate/phosphate region showed significant correlations with PMI. Principal component analysis revealed time-dependent spectral evolution, with PC1 (48.1%) associated mainly with carbohydrate/phosphate variations and PC2 (37.6%) with protein structural changes. Partial least squares regression with two latent variables achieved a cross-validated RMSE of 15.8 h (R^2^ = 0.53) on all 20 known samples. Variable importance analysis identified glycoprotein degradation (1190 cm^−1^) and phospholipid breakdown (736 cm^−1^) as the dominant predictors, with traditional carbohydrate bands playing a secondary role. Predictions for unknown samples ranged from 27.1 to 80.1 h, with five of ten falling within the 90% prediction interval (±20 h) of the available estimates. This study presents a promising PMI estimation model that performed well on unseen samples, even if the sample size represents a methodological limitation that will be addressed in future investigations through larger, more diverse datasets.

## 1. Introduction

The postmortem interval (PMI), the time between death and body discovery, is one of the most challenging issues in forensic medicine and a fundamental question in criminal investigations, as it helps narrow the pool of potential suspects [1,2]. When estimating PMI at a crime scene, forensic pathologists integrate multiple lines of evidence: (i) contextual information from personal belongings, items found near the body, witness testimonies and video footage [1,2]; (ii) conventional postmortem changes (cadaveric modifications) based on the death triad [3,4,5]: algor mortis (body cooling), regarded as the gold standard for estimating early PMI [2,6,7], livor mortis (lividity), and rigor mortis (muscle stiffening); and (iii) entomological data correlating with cadaver decay, which enables PMI classification into immediate, early, or late stages [7,8,9].

Complementary to autopsy, laboratory analyses are conducted on biological samples from the cadaver, including histopathology [10], toxicology [11], and omics sciences (transcriptomics, proteomics, metabolomics, lipidomics) [12,13,14,15,16], to identify consistent and predictive PMI biomarkers. Omics sciences provide factual data that complement and strengthen autopsy reports, while translational research using animal or human models expands fundamental knowledge and aims to narrow PMI estimation. However, even if one were present at a crime scene, establishing the exact time of death beyond reasonable doubt, the legal standard of proof in a court of justice remains exceedingly difficult [17]. Numerous factors introduce variability and limit estimation accuracy, including internal conditions such as cause of death, external factors like environmental conditions, and procedural variables related to sample collection, transport, and laboratory protocols [18].

Vitreous humor (VH) is a viscoelastic hydrogel located in the posterior chamber of the eye. It has a homogeneous matrix structure that helps maintain the shape of the eyeball and consists of two phases: liquid and gel. Water constitutes over 98% of its volume. The structural framework is provided by collagen fibers, predominantly type II, with contributions from types V, VI, IX, and XI [19], arranged in a triple helix rich in glycine, hydroxyproline, and proline. This collagen network ensures both shape and flexibility. Embedded within this matrix are hyalocytes, cells with immune-modulating functions [20], and various non-collagenous components, including glycoproteins such as fibrillin and opticin (the latter playing an anti-angiogenic role) [21]. Glycosaminoglycans (GAGs) are also present. A few are attached to protein cores to form proteoglycans (e.g., those containing chondroitin sulfate and heparin sulfate), while the majority exist as free hyaluronic acid. Hyaluronic acid is unevenly distributed within the eye at concentrations of 140–340 μg/mL [22] and is responsible for the matrix’s stability and viscoelastic properties [23]. In addition to structural elements, VH contains non-structural proteins such as immunoglobulins and complement proteins, as well as lipids, sugars, and inorganic salts. Human eyes have been reported to contain higher collagen concentrations than those of bovine or other species [19].

From a forensic perspective, VH offers several advantages. It lacks many degradative enzymes and is well protected from microorganisms and putrefaction due to its anatomical location [24,25]. Although aging leads to significant compositional changes, such as a decrease in the gel phase and an increase in the aqueous phase (by more than half in individuals over 80 years) [26], the vitreous remains relatively stable after death. Its limited vascularization and the protective role of the blood–retinal barrier contribute to its prolonged postmortem integrity, making it a valuable matrix for PMI estimation [27].

Fourier-transform infrared spectroscopy (FTIR) is a vibrational spectroscopic technique that measures the absorption of infrared radiation by molecular bonds in a sample, generating a spectral “fingerprint” characteristic of its biochemical composition [28]. Unlike conventional infrared methods, FTIR captures all frequencies simultaneously using an interferometer, and the resulting interferogram is converted into a spectrum via Fourier transformation. Attenuated total reflection (ATR) FTIR, a variant that measures the evanescent wave at the sample–crystal interface, offers advantages for biological samples: it requires minimal preparation, is rapid, and avoids fluorescence interference common to Raman spectroscopy [29].

Several spectroscopic techniques, including UV-VIS-NIR [30], FTIR [31,32,33,34], ATR-FTIR [35,36], and Raman spectroscopy [37,38], have been explored for PMI estimation in various tissues and organs. Within this context, VH has emerged as a particularly promising matrix due to its anatomical protection, slower postmortem degradation, and relative independence from immediate environmental conditions. Successful ATR-FTIR studies were conducted both in animal models [39,40,41] and in humans [42,43,44]. PMI is in intense scientific debate once again and benefits from the development of omics sciences that enable a consistent approach [13,18,45,46,47]. Moreover, metabolomic studies have demonstrated that small molecules in biological samples can serve as reliable PMI predictors [48], further supporting the rationale for applying vibrational spectroscopy to the VH matrix.

**Figure 1:**
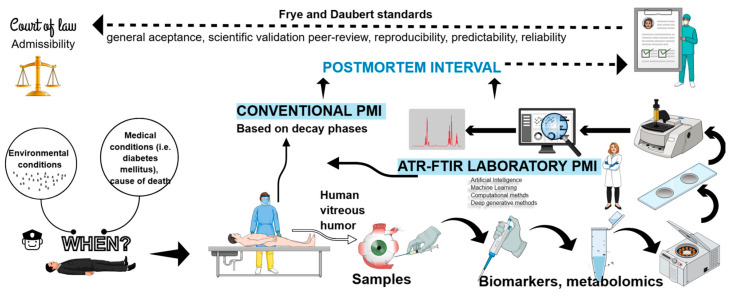
Graphical abstract presenting postmortem interval estimation in legal medicine practice, conditioning factors, present study methodology, postmortem interval estimation using conventional methods and ATR-FTIR method in human sampling (vitreous humor). In a court of law, PMI estimation must meet Fry and Daubert standards.

While previous studies have demonstrated the feasibility of ATR-FTIR spectroscopy combined with multivariate analysis for PMI estimation in VH samples [42,44], these investigations were predominantly conducted under controlled conditions using samples with precisely known PMI. The present study is an exploratory investigation that takes a step toward forensic applications in real-world medico-legal scenarios by considering two sample categories: cases with accurately documented PMI (hospital deaths, n = 20) and cases with estimated PMI (scene deaths, n = 10). By extrapolating spectral and biochemical patterns derived from controlled cases to those with unknown PMI, this study aims to critically evaluate the practical applicability, strengths, and limitations of ATR-FTIR spectroscopy as a supportive tool for forensic PMI estimation, while addressing its biological variability, interpretative limits, and medico-legal relevance (see Figure 1).

## 2. Results

### 2.1. Spectral Changes with PMI

**Figure 2:**
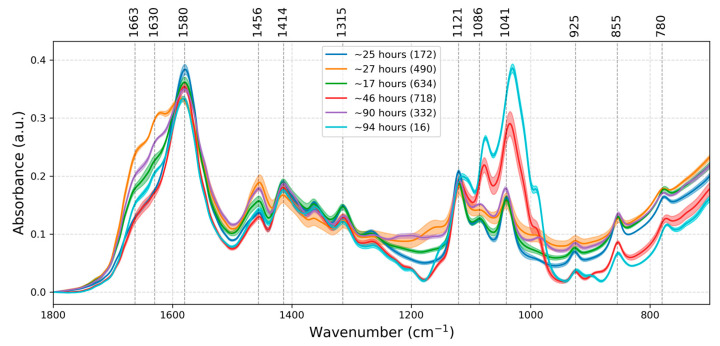
FTIR spectra of samples with known PMI: 172 (~25 h, blue line), 490 (~27 h, orange line), 718 (~46 h, red line), 332 (~90 h, purple line), 16 (~94 h, cyan line) and a sample of unknown PMI: 634 (expected PMI ~17 h, green line). The lines represent mean values over replicates and the shades represent standard deviations, n = 4. The diagnostic peaks are marked with dashed lines and the corresponding wavenumbers are written above.

FTIR spectra of VH samples with known PMI were analyzed to characterize the variation in absorption peaks with PMI. Examples of FTIR spectra measured on samples with different PMI (1800–700 cm^−1^ range) are presented in Figure 2. The main diagnostic peaks previously used in PMI prediction [42,43,44] are highlighted on the figure and are described in detail in Table 1. These peaks reflect contributions from the main biochemical constituents of VH, including proteins and peptides (~1663 cm^−1^, ~1630 cm^−1^, ~1315 cm^−1^), free amino acids (~1414 cm^−1^), lipids (~1456 cm^−1^), lactate (~1121 cm^−1^, ~855 cm^−1^), glucose and other monosaccharides (~1041 cm^−1^), nucleic acids (~925 cm^−1^) or urea, creatinine and uric acid (~1580 cm^−1^). However, due to spectral overlap, these assignments are not exclusive, and many bands (particularly in the 1000–1200 cm^−1^ region) may contain contributions from multiple biomolecule classes.

**Figure 3:**
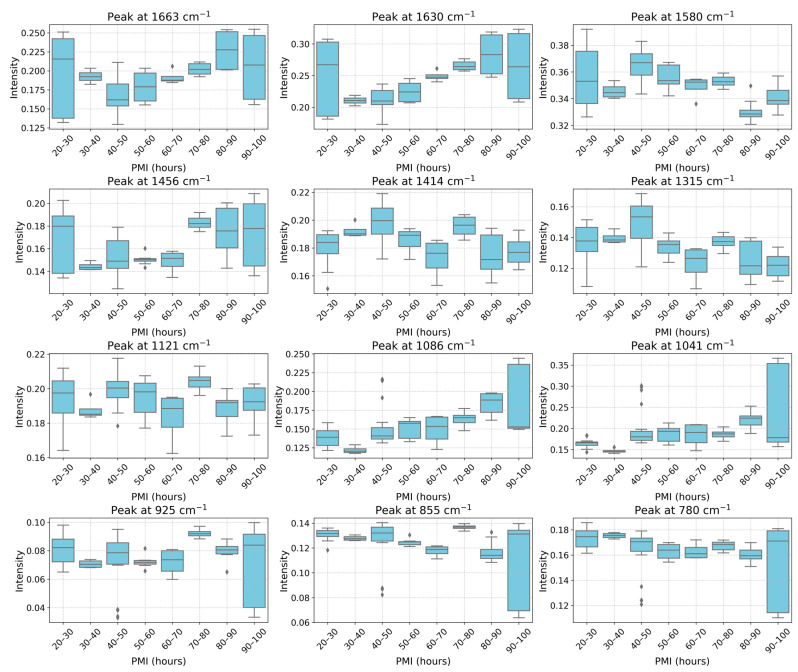
Intensities of diagnostic peaks versus PMI considering 10 h bins in the normalized and corrected spectra profiles. The boxplots are obtained based on the values of peak intensities of replicates (n = 4) of all spectra with PMIs in considered bins.

The variation in diagnostic peaks with PMI is shown in Figure 3. The plots show that results over 10 h PMI bins are mostly consistent, with some exceptions. In the 20–30 h range, the samples show a high variability in absorptions at ~1663, ~1630 and ~1456 cm^−1^. These are associated with protein secondary structure (amide I α-helix and β-sheet content), as well as lipids, proteins, and small metabolites. The outlier in this group is sample 490 (Figure 2), for which the cause of death was thermal trauma from an explosion. Such a condition may induce rapid protein denaturation (shifting α-helix to β-sheet content, altering ~1663/~1630 cm^−1^ ratios), lipid peroxidation (affecting ~1456 cm^−1^ CH_2_ vibrations), and metabolite release [50,51,52]. In addition, the individual had obesity, diabetes, and arterial hypertension, and was hospitalized for 47 days. A more detailed discussion is provided in Supplementary Material S1.

In the 40–50 h PMI range, peaks at ~1086, ~1041 and ~925 cm^−1^ show higher variability. These bands are commonly associated with phosphate-containing compounds, glucose and nucleic acid content. The outlier in this range is sample 718, which shows a marked increase in absorption bands in the 1180–955 cm^−1^ range and a notable decrease in the band at ~925 cm^−1^. The clinical history of sample 718 includes head trauma with subdural hematoma surgery. The observed differences may reflect biochemical alterations due to the underlying condition [53,54,55], the physiological stress response, and substances used during surgical intervention [23,56].

In the 90–100 h PMI range, most absorption bands show elevated variability, particularly those associated with proteins (~1663, ~1630 cm^−1^), phosphate-containing compounds (~1083 cm^−1^), monosaccharides (~1041 cm^−1^), nucleic acids (~925 cm^−1^), and carbohydrates (~855, ~780 cm^−1^). The outlier in this range is sample 16 (cause of death: metastatic pancreatic cancer with acute cerebral stroke and multiple organ failure), which exhibits significant differences in these regions relative to other samples in the same time bin. Acute stroke is known to alter VH composition through breakdown of the blood–ocular barrier, introducing blood-derived proteins (affecting amide bands), inflammatory mediators, and cellular debris [53,54,55]. The spectral profile of sample 16 resembles that of sample 718, despite their different PMIs (Figure 2). This suggests that certain pathological conditions may produce convergent spectral signatures. A more detailed discussion is provided in Supplementary Material S1.

Pearson correlation coefficients between peak intensities and PMI were calculated with 95% confidence intervals. To account for multiple testing, *p*-values were corrected using the Benjamini–Hochberg false discovery rate (FDR) method. As presented in Table 2, five peaks showed moderate to strong correlations (|r| > 0.4) with PMI after FDR correction: 1086 cm^−1^, 1580 cm^−1^, 1315 cm^−1^, 1630 cm^−1^, and 1041 cm^−1^. Additional peaks showed weaker but statistically significant correlations after FDR correction (1663, 1456, 1414, 780, 855 cm^−1^). The bands at 1121 cm^−1^ and 925 cm^−1^ did not reach statistical significance. Previous studies have also noted the importance of these bands for predicting PMI in both human and animal models, with the exception of the ~1580 cm^−1^ band [42,43,44]. In rabbit VH FTIR spectra, the area under the curve centered at ~1586 cm^−1^ decreased in the first 6 h postmortem and remained relatively constant up to 48 h [42]. Here, by analyzing band intensity, we observed a moderate negative correlation with PMI over the 25–98 h range.

### 2.2. Regression Modeling of Data

#### 2.2.1. Principal Component Analysis of VH Spectra

**Figure 4:**
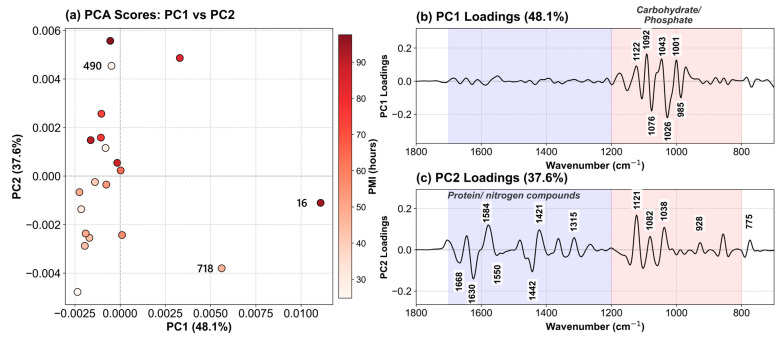
Results of PCA performed on the spectra of VH samples with known PMI considering the 1800–700 cm^−1^ range. (**a**) PC1 vs. PC2 score plot. The PMI is encoded according to the color scale. The outlier samples are labeled. (**b**) PC1 loading spectrum. Red was used to highlight the carbohydrate/phosphate-associated spectral region. (**c**) PC2 loading spectrum. Blue was used to mark the protein-associated spectral region and red was used as described in (**b**). In (**b**,**c**) we labeled the important features on the plots.

PCA was applied to the mean-centered second-derivative spectra (1800–700 cm^−1^) of all 20 VH samples with known PMI. The first two principal components (PCs) explained 85.7% of the total spectral variance (PC1: 48.1%, PC2: 37.6%), which suggests that the two components capture the majority of biochemical variation across samples (Figure 4a). PCA scores show a relative clustering of samples based on PMI: samples with shorter PMI (<48 h) present negative PC1 and PC2 values, while samples with higher PMI cluster at negative PC1 values and positive PC2 values.

The PC1 loading plot (Figure 4b) showed that the most prominent features are concentrated in the 950–1100 cm^−1^ region, which is primarily associated with vibrational modes of carbohydrates and phosphate-containing compounds, though glycoproteins and nucleic acids also contribute in this range. Strong positive loadings are observed at 1092, 1043, and 1001 cm^−1^, corresponding to spectral regions that increase in intensity with advancing PMI. These bands are characteristic of glycogen (1043 cm^−1^) [57,58], phosphate stretching vibrations (1086–1092 cm^−1^), and ribose-phosphate backbone vibrations (1001 cm^−1^) [59,60]. Strong negative loadings appear at 1076, 1026, and 985 cm^−1^, representing spectral features that decrease over time. PC1 is dominated by bands associated with carbohydrate and phosphate vibrations, suggesting that these biochemical domains account for the largest source of spectral variance across samples.

The PC2 loading plot (Figure 4c) is dominated by protein-related features (1500–1700 cm^−1^) represented by strong positive loadings at 1584 cm^−1^ (amide II, coupled C–N stretching and N–H bending), and 1315 cm^−1^ (amide III, C–N stretching and N–H bending), along with strong negative loadings at 1668 cm^−1^ (amide I, α-helical structures), 1630 cm^−1^ (amide I, β-sheet structures) and 1442 cm^−1^ (CH_2_ bending of lipids and proteins). These show that PC2 captures variations in protein secondary structure content. However, moderate positive loadings are observed in the carbohydrate/phosphate region (1121, 1082, and 1038 cm^−1^), indicating that PC2 captures additional, PMI-independent biochemical variation in these spectral domains. This overlap is expected given the biochemical complexity of vitreous humor, where multiple biomolecule classes contribute across broad spectral regions.

The separation of biochemical information into PC1 (time-dependent carbohydrate/phosphate changes) and PC2 (protein variations with additional carbohydrate contributions) represents a validation for our subsequent modeling approach. The substantial contribution of carbohydrate/phosphate-related signals to structured spectral variance across both time-dependent (PC1) and inter-individual (PC2) domains suggests these biochemical markers may be more consistently aligned with PMI progression than protein-derived signals, which are largely confined to PC2.

In what concerns the outliers identified in the previous section, namely samples 490, 718 and 16, we observed that they exhibit particular PC1 and PC2 scores (Figure 4a). Sample 490 (PMI 26.7 h) presents a negative PC1 value, consistent with its early PMI, but exhibits an unusually large positive PC2 value that separates it vertically from other samples in the <48 h range. This suggests that its protein-associated signature is atypical, while its carbohydrate profile reflects appropriate PMI time. Samples 16 (PMI 94.3 h) and 718 (PMI 46.2 h) present the largest positive PC1 values, positioning them outside the cluster of samples with comparable PMI. This suggests that their carbohydrate/phosphate spectral profiles are more characteristic of significantly longer PMIs, possibly related to their respective pathologies. These observations point toward an impact of differential effects of antemortem physiological conditions on distinct biochemical compartments and validate the separation of carbohydrate-driven (PC1) and protein-driven (PC2) information by PCA.

#### 2.2.2. Partial Least Squares (PLS) Regression for PMI Prediction

##### Model Performance Across Multiple Train/Test Splits

To ensure rigorous validation and assess the stability of model performance, the dataset was subjected to five random train/test splits (75/25) with preprocessing parameters derived exclusively from the training set to prevent data leakage. For each split, the training set (15 samples) was used to optimize the number of PLS components via leave-one-out cross-validation (LOOCV), while the test set (5 samples) was held out entirely and used only for final evaluation. EMSC normalization and mean-centering of second-derivative spectra were performed using parameters computed solely from the training set and applied to the test set.

Across the five splits, the optimal number of PLS components ranged between two and 10, with two components being the most frequent choice (two out of five splits; Table S2.1 in Supplementary Material S2). The test set performance showed considerable variability (RMSE range: 6.7–28.8 h; R^2^ range: −0.33 to 0.89), reflecting the heterogeneity of the dataset and the influence of challenging samples (e.g., 16, 490, 718) when they appeared in the test set. The mean ± standard deviation across splits was RMSE = 17.9 ± 7.1 h, R^2^ = 0.41 ± 0.41, and MAE = 13.7 ± 5.2 h. Detailed results are provided in Supplementary Material S2.

##### Final PLS Model Calibrated on All Known Samples

Based on the most frequent optimal component count across the five splits, a final PLS model with two latent components was calibrated on all 20 known samples. LOOCV was used to obtain an unbiased estimate of predictive performance. The model achieved a cross-validated RMSE of 15.82 h, an R^2^ of 0.531, and a mean absolute error of 12.27 h (Table 3, Figure S2.1 in Supplementary Material S2). Prediction errors across the 20 known samples ranged from 0.1 to 36.1 h, with 14 samples classified as reliable (error < 15.8 h), 4 as borderline (error in 15.8–31.6 h range), and 2 as problematic (samples 490 and 718, with errors of 36.1 h and 33.3 h, respectively; Table S2.2, Figure S2.1). A detailed analysis of sample reliability and error distribution is provided in Supplementary Material S2.

##### Interpretation of PLS Model: Standardized Regression Coefficients and Variable Importance

**Figure 5:**
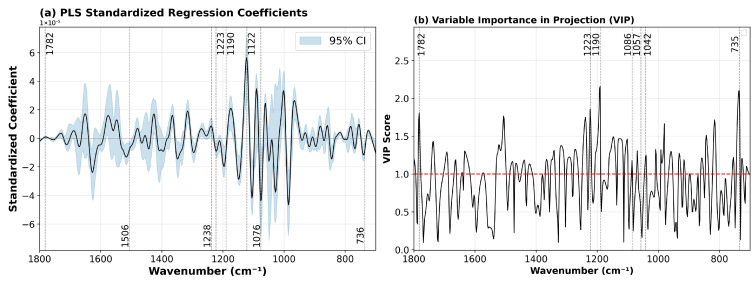
Interpretation of the PLS regression model (2 components). (**a**) Standardized regression coefficients for the final model. Shaded areas represent 95% bootstrap confidence intervals. Key spectral regions are annotated with their approximate wavenumbers. (**b**) Variable importance in projection (VIP) scores. The horizontal red dashed line indicates the VIP = 1.0 threshold; wavenumbers above this line are considered significant predictors. Selected VIP peaks are labeled.

The standardized regression coefficients (Figure 5a) reveal the direction and magnitude of association between each spectral region and PMI, after accounting for correlations among wavenumbers [61,62]. Positive coefficients indicate bands that increase in intensity with advancing PMI, while negative coefficients indicate bands that decrease.

The largest positive coefficients were observed in the 1120–1126 cm^−1^ region, peaking at 1122.5 cm^−1^ (coefficient = 5.6 × 10^−5^). This region is assigned to C–O–C stretching vibrations of glycoproteins, small carbohydrates and phosphodiesters [63]; however, it may also receive contributions from other phosphate-containing compounds and nucleic acids. The strong positive coefficient indicates progressive accumulation or structural rearrangement of glycoprotein components with PMI, consistent with postmortem degradation of the vitreous matrix.

Strong negative coefficients were observed in the 1070–1080 cm^−1^ region, particularly at 1076 cm^−1^ (−4.4 × 10^−5^) and 1074 cm^−1^ (−4.3 × 10^−5^). This region is associated with phosphate stretching vibrations (PO_2_^−^) and C–O stretching of carbohydrates [63]. The negative sign suggests a decrease in these components over time, consistent with the depletion of energy reserves (glycogen) and breakdown of phosphate-containing compounds during the postmortem interval.

The lipid ester region at 1782–1784 cm^−1^ showed positive coefficients (1.8 × 10^−6^ to 8.2 × 10^−7^), indicating accumulation of lipid oxidation products with PMI [64]. The phospholipid region at 735–740 cm^−1^ exhibited strong negative coefficients (up to −1.1 × 10^−5^), consistent with progressive membrane degradation [65].

The amide III/nucleic acid region (1220–1240 cm^−1^) [63,66] showed mixed signs: bands at 1223 and 1236 cm^−1^ were negative, while bands at 1238 and 1240 cm^−1^ were positive. This pattern likely reflects competing processes represented by the loss of native protein structure (negative) alongside accumulation of degradation products (positive) and nucleic acid breakdown.

The aromatic amino acid region (1504–1506 cm^−1^) [67] showed negative coefficients (−1.7 × 10^−5^ to −1.5 × 10^−5^), indicating loss of tyrosine and phenylalanine signals, consistent with protein degradation and release of free amino acids.

Variable importance in projection (VIP) scores (Figure 5b) rank the overall contribution of each wavenumber to the PLS model [62,68]. Wavenumbers with VIP > 1.0 are considered significant predictors.

The highest VIP scores were observed at 1190 cm^−1^ (VIP = 2.16) and 1192 cm^−1^ (VIP = 2.13), confirming the dominant role of the glycoprotein, small carbohydrate and phosphodiester region in PMI prediction. The phospholipid region at 735–739 cm^−1^ ranked next (VIP = 2.02–2.10), highlighting the importance of membrane degradation processes. The lipid ester region at 1782–1784 cm^−1^ also showed high VIP scores (1.72–1.81), underscoring the contribution of lipid oxidation.

The amide III/nucleic acid region (1220–1240 cm^−1^) exhibited VIP scores between 1.67 and 1.86, indicating significant contributions from protein secondary structure changes and nucleic acid degradation. The tyrosine region at 1504–1506 cm^−1^ also ranked highly (VIP = 1.73–1.76), reflecting the importance of aromatic amino acid changes.

Traditional carbohydrate/phosphate bands at 1086 cm^−1^ and 1041 cm^−1^ were not among the top 20 VIP scores. This indicates that, while they correlate with PMI in univariate analysis, their predictive information is largely shared with other bands in the model, consistent with the overlapping nature of spectral features in this region.

In summary, the combined analysis of standardized regression coefficients and VIP scores reveals that glycoprotein degradation (1190–1192 cm^−1^), phospholipid membrane breakdown (735–739 cm^−1^), and lipid oxidation (1782–1784 cm^−1^) are the dominant biochemical processes captured by the PLS model, while traditional carbohydrate markers play a secondary, supportive role.

#### 2.2.3. Prediction of PMI for Unknown Samples Using the PLS Model

The final two-component PLS model, calibrated on all 20 known samples, was applied to the ten samples with unknown or uncertain PMI. Predictions ranged from 27.1 to 80.1 h, with prediction uncertainty reported using percentiles of absolute errors from the final model (50th percentile = 7 h; 75th percentile = 16 h; 90th percentile = 20 h; Table 4). The 95% confidence interval (±36.1 h) is provided for reference but is not used as the primary uncertainty measure due to its width and the non-normal residual distribution (Shapiro–Wilk test, *p* = 0.047).

The agreement between PLS-predicted and estimated PMI was assessed based on the 90% confidence interval (±20 h), which corresponds to the 90th percentile of absolute prediction errors from the final model. Estimates falling within this interval were classified as consistent, while those falling outside were considered discrepant. By applying this rule, five samples were classified as consistent: 647 (~12 h, predicted 29.6 h), 723 (~19 h, predicted 28.9 h), 467 (~33 h, predicted 27.1 h), 1247 (~33 h, predicted 39.6 h), and 598 (~35.5 h, predicted 39.2 h). Among these, samples 467, 1247, and 598 were within the 50% confidence interval (±7 h), while sample 723 was within the 75% interval (±16 h) and sample 647 was within the 90% interval (±20 h).

Four samples were classified as discrepant, with estimates falling outside the 90% confidence interval: 634 (~17 h, predicted 37.7 h; ~1 h below lower bound), 637 (~21 h, predicted 48.5 h; ~9 h below lower bound), 643 (~20 h, predicted 54.6 h; ~14 h below lower bound), and 205 (~15 h, predicted 67.0 h; ~30 h below lower bound). Sample 205 (PMI ~15 h, hypothermia) was shown to present a particular spectral shape [69,70], as revealed by the hierarchical clustering of FTIR spectra presented in Supplementary Material S1. The sample clusters with cases 718 (PMI 46.2 h, head trauma and subdural hematoma surgery) and 16 (PMI 94.3 h, pancreatic cancer, acute cerebral stroke). Despite their differences in PMI, these samples share similar spectral features that likely reflect common biochemical pathways activated by blood–ocular barrier disruption (samples 16 and 718) and hypothermia-induced metabolic shifts (sample 205), which may explain the discrepant prediction for sample 205 in the PLS model (Table 4).

The notable exception was sample 641 (estimated ~179 h, predicted 80.1 h; ~79 h above upper bound of 90% CI). In this case, the predicted PMI is consistent with the slowed biochemical degradation due to the longer storage at low temperature [71]. FTIR spectroscopy captured the biochemical signature of the sample that appears decoupled from chronological PMI. This observation reinforces that FTIR spectroscopy detects biochemical age rather than chronological time alone. This distinction has important implications for forensic casework where temperature history may be unknown or variable.

#### 2.2.4. Binary Classification of Samples for Forensic Triage

While continuous PMI estimation provides detailed temporal resolution, forensic casework often benefits from rapid categorization into broad postmortem intervals for initial triage or when only a binary decision is required. To address this practical need, we evaluated several classification methods to distinguish early (≤48 h) from late (>48 h) decomposition stages, including Linear Discriminant Analysis (LDA), Quadratic Discriminant Analysis (QDA), Support Vector Machines (SVM) with linear and radial basis function (RBF) kernels, and Random Forest. Model performance was assessed using LOOCV.

The results in Table 5 show that Random Forest is the classifier that achieved the highest cross-validated accuracy of 70.0% (14/20 samples correctly classified), outperforming LDA (60.0%), SVM (60.0%), and QDA (45.0%). The confusion matrix for Random Forest showed that late PMI samples (>48 h) were very well detected (recall = 92%, 11/12 correctly classified), while early PMI samples (≤48 h) were more challenging to classify (recall = 38%, 3/8 correctly classified). However, when the model predicted an early sample, it was correct 75% of the time (precision = 0.75).

In what concerns the misclassified samples, the five early samples misclassified as late (490, 371, 152, 1148, 718) included cases with documented clinical confounders (490: thermal burn; 718: head trauma) and samples with PMI values approaching the 48 h threshold (1148: 43.6 h). The only late sample misclassified as early was 455 (PMI 49.0 h), which lies just above the threshold. This pattern is consistent with the continuous nature of postmortem biochemical changes and the arbitrary nature of any dichotomous threshold.

When applied to the 10 unknown samples, Random Forest correctly classified 8 of 10 samples (80%) based on available PMI estimates (Table 6). Confidence scores ranged from 0.52 to 0.87, with correctly classified samples showing moderate to high confidence (0.52–0.83). The two misclassifications were sample 643 (estimated ~20 h, predicted late with 52% confidence) and sample 641 (refrigerated, predicted late with 87% confidence). Notably, the refrigerated sample 641 was classified as late with high confidence, while sample 643 showed lower confidence for its late classification.

The top wavenumbers contributing to the Random Forest model included features at 1512 (importance 0.0342), 1529 (importance 0.0333), 1510 (importance 0.0326), and 1516 cm^−1^ (importance 0.0304) (tyrosine/phenylalanine region); 1776 (importance 0.0270) and 1778 cm^−1^ (importance 0.0207) (lipid esters); and 1007 cm^−1^ (importance 0.0192) (carbohydrates; also contains contributions from phenylalanine and nucleic acids), further highlighting the importance of aromatic amino acids, lipid oxidation, and carbohydrate metabolism as key discriminators between early and late postmortem intervals.

#### 2.2.5. Comparison of Regression Methods for PMI Prediction

To contextualize the performance of the PLS regression model and assess the potential influence of outliers, we conducted a systematic comparison using five regression methods with distinct underlying principles: ordinary least squares (OLS—baseline linear regression), PLS (dimension reduction maximizing covariance with PMI), Huber regression (robust M-estimator reducing influence of outliers), Theil–Sen Regression (non-parametric median-based slope estimator) and RANSAC (random sample consensus with automatic outlier detection). All models were applied to the same preprocessed spectra (mean-centered second derivatives) and evaluated using LOOCV. Performance was assessed on four datasets: (i) all 20 known samples, (ii) a subset of samples excluding the samples that appeared as outliers in the intensity of diagnostic peak analysis (samples 490, 718 and 16), (iii) a subset of samples excluding those flagged as problematic by the PLS model (samples 490 and 718), and (iv) a forensically relevant subset with PMI < 72 h (n = 13).

On the full dataset (n = 20), PLS regression with two components achieved an RMSE of 15.82 h and R^2^ of 0.531, comparable to OLS (RMSE = 15.57 h, R^2^ = 0.545) (Table 7). Theil–Sen produced identical results to OLS (RMSE = 15.57 h, R^2^ = 0.545). Theil–Sen is a theoretically robust model due to its median-based nature (breakdown point of 29.3% [72]). On our dataset, the Theil–Sen algorithm fails to compute its full median-based estimator (number of iterations = 0 and number of subpopulations = 1) and defaults to ordinary least squares, producing results identical to OLS. Given this aspect, Theil–Sen results will not be discussed further. RANSAC performed slightly worse (RMSE = 15.91 h, R^2^ = 0.525), while Huber regression failed completely on this dataset, yielding negative R^2^ values of -0.110, indicative of model misspecification or convergence issues with high-dimensional spectral data. These results indicate that with two components, the spectra–PMI relationship captured by PLS is essentially linear, achieving performance comparable to simpler methods on the complete heterogeneous dataset (RMSE = 15.82 h vs. 15.57 h for OLS).

When samples with documented clinical confounders (490: thermal burn; 718: head trauma; 16: cancer/stroke) were removed, leaving 17 samples, all methods improved substantially. OLS and RANSAC showed dramatic improvements, with RMSE decreasing by approximately 4.6–5.6 h (OLS: from 15.57 to 10.93 h; RANSAC: from 15.91 to 10.93 h) and R^2^ increasing to 0.75–0.75. PLS also improved, with RMSE decreasing from 15.82 to 12.27 h and R^2^ increasing from 0.531 to 0.688. Notably, on this cleaner dataset, OLS and RANSAC outperformed PLS (RMSE = 10.93 h and 10.93 h, respectively, vs. 12.27 h for PLS), suggesting that when the data are more homogeneous, simpler methods can capitalize on the simplified structure.

We obtained very interesting results when only the two samples flagged as problematic by the two-component PLS model (490 and 718) were removed, while retaining sample 16 (cancer/stroke), resulting in a dataset of 18 samples. Under this condition, PLS maintained stable performance (RMSE = 12.44 h, R^2^ = 0.705, MAE = 10.01 h), slightly improved compared to the results when excluding clinical confounders. In contrast, OLS and RANSAC collapsed, with RMSE soaring to 18.17 h and 18.19 h, respectively, and R^2^ decreasing to 0.370 and 0.369. This divergence demonstrates that PLS was the only model capable of extracting predictive information from the complex, clinically confounded samples (such as sample 16) that simpler linear methods cannot leverage. Sample 16, despite its unusual spectral appearance and cancer/stroke history, contains valuable biochemical information that can be harnessed by multivariate methods like PLS regression.

**Figure 6:**
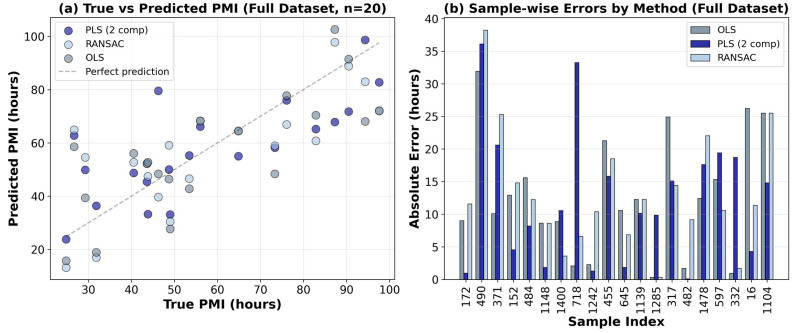
Comparison of regression methods for PMI prediction. (**a**) True versus predicted PMI for PLS, OLS and RANSAC. (**b**) Sample-wise absolute errors for PLS, OLS and RANSAC.

Figure 6a presents true versus predicted PMI for the three best-performing methods (PLS, OLS and RANSAC). PLS shows the closest alignment with the ideal diagonal line, particularly for samples in the 40–80 h range, while RANSAC exhibits greater scatter but captures the overall trend. OLS shows intermediate behavior, with predictions closely following the diagonal but with some deviation at higher PMI values. Figure 6b provides a sample-wise comparison of absolute errors for OLS, PLS, and RANSAC. PLS consistently achieves lower errors across most samples, with particularly notable improvements for samples 172, 1148, 645, and 482. The occasional spikes observed for OLS and RANSAC, as seen with samples 490 and 16, underscore their vulnerability to clinically complex cases, while PLS maintains more stable performance.

When the analysis was restricted to the 13 samples with PMI lower than 72 h (a forensically relevant range), all methods showed decreased performance, with negative R^2^ values indicating that simple linear models failed to capture the underlying structure (Table 7). Huber regression emerged as the best performer in this subset (RMSE = 13.04 h, MAE = 10.73 h), suggesting that robust M-estimation may be advantageous when the PMI range is restricted and non-linearities become more apparent. PLS performed poorly in this range (RMSE = 20.46 h, R^2^ = −2.102), indicating that the two-component model optimized for the full PMI range is not well-suited for this narrow sub-range. This underscores the challenge of PMI prediction with limited sample sizes and restricted ranges, and highlights the need for larger datasets spanning the full temporal spectrum.

As shown in Figure 6b, the two-component PLS model identified 490 as the most problematic sample (error = 36.1 h), consistent with its extreme RANSAC error (38.3 h) and OLS error (30.7 h), confirming its atypical influence across all methods. In contrast, sample 16, despite its complex clinical history (cancer/stroke), was handled effectively by PLS (error = 4.3 h) and showed only moderate errors in RANSAC (12.5 h) and OLS (28.5 h). Sample 718 showed variable behavior: problematic for PLS (error = 33.3 h), but well-predicted by RANSAC (error = 7.3 h) and OLS (error = 0.9 h), highlighting the varying sensitivity of different regression methods to specific outliers.

These results demonstrate that no single method is universally optimal. Method selection should be guided by the characteristics of the available data and the specific forensic question. For heterogeneous casework including diverse causes of death, the two-component PLS model offers a robust, parsimonious solution that can leverage information from complex clinical cases (such as sample 16) while maintaining stable performance. For cleaner datasets without such complexity, simpler methods like OLS or RANSAC may prove superior. The superior performance of Huber regression in the restricted PMI range (<72 h) suggests that different modeling approaches may be required for different forensic contexts, and that method selection should be tailored to the specific PMI range of interest.

## 3. Discussion

### 3.1. Compliance with Daubert and Frye Standards—Forensic Admissibility Framework

Postmortem interval is always an important problem to solve for medico-legal and judicial purposes. Medico-legal causality implies not only the cause of death and events in a temporal link but also assessment of postmortem interval. Admissibility in a court of law [17] is always tested using Daubert standards (checking scientific validity, reproducibility, predictability and reliability of clinical and laboratory data and postmortem interval estimation) [73] and Frye Standard (based on the general acceptance of the evidence) [74].

Our study was designed to address the admissibility criteria through a systematic, forensic-oriented approach: (i) scientific validity: we incorporated both known and unknown PMI cases, validating spectral patterns derived from controlled cases against real-world forensic scenarios; (ii) reproducibility: we performed a rigorous cross-validation and independent test set evaluation, along with interpretable biochemical marker analysis; (iii) predictability: the estimation of PMI for ten unknown samples was largely consistent with available case information; (iv) reliability: we performed multiple complementary analyses to validate the PLS model, and we transparently reported the error rates and limitations. This positions ATR-FTIR spectroscopy as a scientifically defensible adjunctive tool for PMI estimation in forensic practice.

### 3.2. Comparison with Previous Studies and Novelty of the Present Approach

Previous studies conducted on human [44] and rabbit [42] VH samples have shown that ATR-FTIR measurements assisted by chemometric analysis and machine learning can be used to predict the PMI of a sample. The study of Zhang et al. [42] provides a proof-of-concept for ATR-FTIR-based PMI estimation under highly controlled experimental conditions, using 72 rabbits with uniform PMIs (0–48 h). They achieved a good predictive accuracy with an ANN model (R^2^ = 0.983, RMSE = 2.018 h) and identified the 1313 and 925 cm^−1^ bands as highly correlated with PMI.

Our study also identified the contribution of the 1315 cm^−1^ region (amide III) to PMI prediction (VIP = 1.37, positive regression coefficient = 1.92 × 10^−5^), which represents an important cross-species validation. This finding suggests that protein degradation patterns in VH could follow a conserved biochemical pathway across mammals. Still, the 925 cm^−1^ band (nucleic acids, with contributions from carbohydrates and phosphodiesters) showed strong correlation in rabbits but minimal contribution in our human PLS model (VIP = 1.01, negligible coefficient = 1.10 × 10^−6^). This discrepancy likely reflects species-specific differences in postmortem nucleic acid degradation rates or the confounding effects of diverse human causes of death, factors absent in controlled animal studies.

In the case of human VH, Notarstefano et al. [44] established the methodological feasibility, reporting >80% classification accuracy using PLS-DA. They identified discriminant peaks, including those at 1120, 1630, and 1585 cm^−1^, which they attributed to protein degradation, amino acid deamination, lactate accumulation, and hyaluronic acid modifications.

Our results independently validate several of these observations. The glycoprotein/phosphodiester region emerged as the dominant predictor in our PLS model, with the highest VIP scores at 1190 cm^−1^ (VIP = 2.16) and 1192 cm^−1^ (VIP = 2.13), highlighting the importance of glycoprotein degradation, a dimension less emphasized in previous studies. The 1630 cm^−1^ β-sheet band showed a VIP of 1.27 and a negative coefficient (−2.23 × 10^−5^), consistent with progressive protein denaturation. The 1580 cm^−1^ amide II band exhibited a VIP of 0.78 and a positive coefficient (7.61 × 10^−6^), reflecting changes in protein secondary structure.

The band at 1121 cm^−1^, which corresponds to the 1120 cm^−1^ peak identified by Notarstefano et al. [44], showed a VIP of 1.23 and a negative coefficient (−1.53 × 10^−6^), confirming its contribution while indicating that its role is secondary to the dominant glycoprotein and phospholipid regions identified by our model. This region additionally contains overlapping signals from glycoproteins, phosphodiesters, and small carbohydrates. Notably, our data-driven VIP analysis also identified the 734–739 cm^−1^ phospholipid region (VIP = 2.02–2.10) and the 1782–1784 cm^−1^ lipid ester region (VIP = 1.72–1.81) as highly influential predictors, highlighting the importance of membrane degradation and lipid oxidation, processes not previously emphasized in human VH studies.

The agreement with previous findings, despite differences in dataset composition and analytical pipelines, supports the robustness of these spectral features as PMI biomarkers.

However, our study extends the previous foundational work in several critical directions. We adopted a forensic-oriented design incorporating both cases with precisely known PMI (hospital deaths) and cases with unknown or presumed PMI (scene deaths). In this way we directly addressed the gap between laboratory-controlled conditions and real-world medico-legal scenarios. The inclusion of cases with unknown PMI introduces uncertainty but provides a more realistic assessment of the strengths and limitations of spectroscopic methods in forensic settings. Rather than representing a methodological weakness, this approach highlights the boundaries within which ATR-FTIR spectroscopy may support, but not replace, classical medico-legal evaluation.

Our dataset is fully disclosed, with indications of age, cause of death and related comorbidities. We performed a detailed outlier analysis revealing that clinically complex cases (490—thermal burn; 718—head trauma) impair predictive performance, while visually atypical but structurally informative samples (16—cancer/stroke) are effectively handled by PLS, underscoring the method’s robustness to biological heterogeneity. In addition, the observation that a refrigerated sample (641) was predicted at 80.1 h despite an estimated PMI of ~179 h demonstrates that FTIR detects biochemical age rather than chronological time, a distinction with important implications for forensic casework where temperature history is unknown. In this way, our study addresses not only the algorithmic performance of PMI prediction models but also considers the biological and medico-legal interpretability of spectral changes. This interpretative framework is essential for forensic application, as medico-legal conclusions cannot rely exclusively on algorithmic outputs but must be supported by biologically plausible mechanisms and expert judgment.

It should be noted that our model was calibrated on samples with PMI ranging from 24.8 to 97.6 h (approximately 1 to 4 days), corresponding to early to intermediate postmortem intervals. The model is therefore intended for use within this range, and its applicability to shorter or longer intervals remains to be validated. Environmental factors such as temperature and decomposition conditions may further influence spectral signatures, as evidenced by the refrigerated sample (641), which showed a predicted PMI of 80.1 h despite an estimated chronological age of 179 h.

### 3.3. Methodological Considerations and Limitations; Future Perspectives

Despite the above-mentioned advances, we acknowledge several methodological limitations that should be considered when interpreting the results. The modest sample size (20 known PMI cases, 10 unknown) limits statistical power and generalizability. This supports the need for validation in larger, more diverse cohorts. The heterogeneity of causes of death, while reflective of real forensic practice, introduces biological variability that may influence vitreous humor biochemical profiles and complicates the identification of universal PMI biomarkers. 

A specific challenge arises from the design choice to calibrate the model on hospital deaths (n = 20) and apply it to scene deaths with estimated PMI (n = 10). While this reflects real forensic scenarios, it introduces the possibility that systematic biochemical differences between hospital and scene deaths may confound predictions. To assess this, we deliberately included diverse causes of death in both cohorts, including violent deaths (thermal burns, traumatic brain injury, drug intoxication, hanging, hypothermia, fall from height) and non-violent deaths (septic shock, cancer). Among scene death cases, predictions were generally consistent for drug intoxication (647), hypothermia (723), choking (467), septic shock (1247), and hanging (598), while discrepancies were observed for fall from height (634, 643), thermal burns (637), and one hypothermia case (205). Notably, two hypothermia cases showed divergent outcomes: one (723) was well-predicted (within 75% CI), while the other (205) was discrepant, suggesting that factors beyond cause of death (e.g., agonal period, body temperature at discovery, or individual physiological variation) may modulate the spectral signature. These observations highlight that while the model performed well for certain scene death cases, the small number of samples per cause of death impairs definitive conclusions about the effect of specific pathologies. Larger datasets are needed to determine whether cause-specific models are required or whether the current approach can be generalized with appropriate confidence intervals.

Additionally, the lack of detailed environmental data (especially temperature) impaired the development of temperature-corrected models. The identification of sample 641 as a refrigerated case showing a discrepancy between the PMI and the biochemical age demonstrates the potential importance of such environmental factors.

For context, conventional PMI estimation methods have well-documented limitations. Body temperature-based methods, considered the gold standard for early PMI, achieve accuracies of ±2–3 h within the first 12 h, but errors increase substantially with longer intervals [75]. Forensic entomology can provide estimates with errors of ±12–48 h depending on species and environmental conditions [7,8,9]. Omics-based approaches (metabolomics, proteomics) have reported RMSE values of 10–20 h under controlled laboratory conditions [76]. Our RMSE of 15.8 h is therefore comparable to existing laboratory-based methods, albeit with a wider prediction interval (±36.1 h) that reflects the heterogeneity of our dataset and the challenges of real-world forensic conditions. The 90% prediction interval (±20 h) provides a more practical measure of uncertainty for forensic triage, correctly classifying 5 of 10 unknown samples within this range.

Future research should prioritize (i) expanding datasets across multiple centers to capture broader demographic and environmental diversity; (ii) developing cause-specific or context-adjusted models that account for variables such as cause of death, agonal period, and storage conditions; (iii) integrating spectroscopic findings with complementary omics approaches (metabolomics, proteomics) to elucidate underlying biochemical mechanisms; and (iv) validating the approach on portable FTIR devices for potential on-site application at crime scenes.

In conclusion, this study confirms that ATR-FTIR spectroscopy of vitreous humor is a promising adjunctive tool for PMI estimation, with demonstrated applicability to the diverse, imperfect conditions of real forensic practice. The methodological framework established here—incorporating both known and unknown PMI cases, rigorous validation, and interpretable biochemical markers—provides a foundation for future large-scale studies aimed at integrating spectroscopic PMI estimation into routine medico-legal casework.

## 4. Materials and Methods

### 4.1. Case Selection

The study took place in the National Institute of Legal Medicine Mina Minovici Bucharest (NILM). We selected 30 cases that were divided into two different groups (described in detail in Supplementary Material S3). For all these cases, a full autopsy, external and internal, was performed at the official request. All cases are unidentified persons.

Informed consent is N/A (all bodies are unidentified, postmortem interval estimation is officially requested in all officially legal autopsies, and all legal autopsies are mandatory when the judicial order is issued according to the Romanian Penal Code, art. 185). The study has ethical clearance (3000/2024 NILM). The sample were taken at the autopsy in NILM and analyzed at the molecular biology laboratory with FTIR facilities at the Dept. of Anatomy, Animal physiology and Biophysics, University of Biology, University of Bucharest, Romania. A research protocol including a biohazard sampling protocol (transport conservation, etc.) was established between the two above-mentioned institutions.

The assignment of samples to known versus unknown PMI cohorts was guided by temporal documentation. Cohort 1, known PMI (Table 8 and Table S3.1), comprised 20 hospital deaths with reliably documented times of death and PMI values spanning a continuous range from 24.8 to 97.6 h.

Cohort 2, unknown PMI (Table 7 and Table S3.2), included 10 samples: 9 scene deaths with only estimated PMI, and one hospital death (sample 641; PMI 179.28 h) that was excluded from the known PMI cohort. Sample 641 presents a ~79 h temporal discontinuity from the next highest PMI (97.58 h). Including this sample in the known PMI cohort would have required the model to extrapolate across an uncharacterized region, potentially distorting the degradation trajectory estimated from the more densely sampled 25–100 h range. What is more, a particularity of sample 641 is the prolonged storage under refrigeration due to objective reasons independent of study design. By assigning sample 641 to the unknown cohort, we could directly test the model’s ability to estimate the PMI under conditions in which the refrigeration delays the natural body decomposition.

### 4.2. VH Sample Collection

Samples of VH were taken from both eyes, right eye (RE) and left eye (LE), at the time of autopsy. Cadavers were kept in a refrigerator (rectal temperature 5 °C), and ambient temperature was 20 °C. Cadaver eyes were opened, and sampling was performed with a syringe: 0.5 cm deep, 20-gauge puncture of the eyeball at an angle of 45 degrees in the outer superior quadrant of the eyeball, gently removing 1 mL of its content. The VH samples from RE and LE were placed in sterile Eppendorf tubes and frozen at −80 °C. Transport occurred according to the protocol in special containers on ice immediately after removal of the sample.

### 4.3. Sample Processing and ATR-FTIR Measurements

After thawing, 100 µL of each sample was mixed with 100 µL methanol (Sigma-Aldrich, St. Louis, MO, USA) to remove proteins [48]. The tubes were centrifuged at 14,000 rpm for 10 min. From the supernatant, 50 µL from each sample was transferred to pre-cleaned glass coverslips and kept in a temperature-controlled oven at 40 °C until fully dry. The resulting dried films were carefully detached using a clean surgical knife and transferred to the ATR crystal of an FTIR Bruker Tensor 27 spectrophotometer (Bruker Optik GmbH, Ettlingen, Germany). This indirect drying approach was developed to address challenges with direct ATR drying, including prolonged drying times, difficulties removing dry samples from the crystal surface and toxicity building due to methanol release in the laboratory. Spectra were recorded in the 4000–400 cm^−1^ range, with a 4 cm^−1^ resolution, during a collection time of 1 min.

### 4.4. Data Analysis

Data analysis was performed in Python (v3.11.7; Python Software Foundation, Wilmington, DE, USA) using the NumPy (version 1.21.0), pandas (version 1.3.0), SciPy (version 1.7.0), and scikit-learn (version 1.0.2) libraries.

#### 4.4.1. Spectral Processing

All FTIR spectra (1800–700 cm^−1^ frequency range) were preprocessed using a three-step pipeline: Extended Multiplicative Signal Correction (EMSC), second-derivative transformation, and mean-centering. Critically, the reference spectrum for EMSC and the mean for centering were derived from different sample sets depending on the analysis, as detailed below.

##### Extended Multiplicative Signal Correction (EMSC)

Raw spectra were normalized using EMSC to minimize non-chemical variability (scaling and baseline effects) while preserving biochemical information [77]. The mean spectrum of the 20 known PMI samples served as the EMSC reference for all analyses, including peak intensity analysis, PCA, the final PLS model, binary classification, and the comparative regression analysis. The only exception was during PLS model development, where the known PMI spectra were split into a training set (15 samples) and a test set (5 samples). In this case, EMSC normalization was performed using the mean spectrum of the training set to prevent data leakage. A first-order polynomial baseline was included in the EMSC model to account for wavelength-dependent scattering effects.

##### Second-Derivative Transformation

Following EMSC, second derivatives were calculated using a Savitzky–Golay filter with a 15-point window and a third-order polynomial [78]. This transformation enhances spectral resolution, minimizes residual baseline variations, and resolves overlapping bands. Second derivatives were applied per spectrum and do not involve data pooling.

##### Mean-Centering

The final preprocessing step was mean-centering [79], applied separately for different analytical purposes. For PCA, final PLS model, binary classification and comparative regression analysis, mean-centering was performed using the global mean of the 20 known PMI samples. This ensures that all analyses reflect the full spectral variability of the known dataset. For PLS model development (component optimization and test set validation), the dataset was split into a training set (15 samples) and a test set (5 samples). The mean spectrum was computed exclusively from the training set and subtracted from both the training and test sets. This prevents data leakage, ensuring that test set predictions are unbiased. For unknown samples, mean-centering was performed using the global mean of the 20 known PMI samples, consistent with the final model calibration.

#### 4.4.2. Peak Intensity Analysis

We analyzed the intensity of peaks usually addressed in similar studies of PMI prediction based on VH [42,43,44] (Table 1), peaks that we call “diagnostic peaks” throughout the text. We preferred to address intensities because calculating the area under some bands and especially under some shoulders in our spectra was challenging. By this approach, we aimed to obtain a more reproducible quantification across all samples and operators. For the analysis, spectra were offset corrected at 1800 cm^−1^; therefore, reported peak intensities reflect relative spectral variations rather than absolute absorbance values.

Correlations between peak intensities and PMI were assessed using Pearson correlation coefficients. For Pearson correlations, 95% confidence intervals were calculated using Fisher’s z-transformation [80]. To control for false discovery rate due to multiple testing (12 peaks), *p*-values were corrected using the Benjamini–Hochberg method [81]. Correlations with FDR-corrected *p* < 0.05 were considered statistically significant.

#### 4.4.3. Principal Component Analysis (PCA)

PCA [82] was applied as an unsupervised exploratory tool to visualize the natural clustering of samples, identify potential outliers, and examine the main sources of spectral variance in relation to PMI [83]. PCA was performed on the mean-centered second-derivative spectra (1800–700 cm^−1^) of all 20 known samples. Score plots were examined for systematic trends with PMI, and loading plots were inspected to identify the spectral regions contributing most to each PC.

#### 4.4.4. Partial Least Squares Regression (PLS)

PLS regression was employed to develop a quantitative predictive model for PMI estimation [62,68]. PLS extracts latent variables that maximize covariance between the spectral matrix X and the response vector y (PMI), making it well-suited for high-dimensional, collinear spectral data with limited sample sizes [61].

##### Model Validation Using Multiple Train/Test Splits

To assess model stability, the dataset was subjected to 5 random train/test splits (75%, n = 15/25%, n = 5). For each split, preprocessing parameters were derived exclusively from the training set. The optimal number of PLS components was determined via leave-one-out cross-validation (LOOCV) on the training set, selecting the component number that minimized the cross-validated root mean squared error of prediction (RMSECV) [84]. Test set performance was evaluated once per split.

##### Final Model Calibration

Based on the most frequent optimal component count across the 5 splits, a final PLS model with two latent components was calibrated on all 20 known samples. LOOCV was used to obtain an unbiased estimate of predictive performance. Model performance was assessed using root mean squared error (RMSE), mean absolute error (MAE), and the coefficient of determination (R^2^).

##### Spectral Interpretation

To identify the spectral regions most influential in the PLS model, variable importance in projection (VIP) scores were calculated. VIP scores summarize the contribution of each wavenumber weighted by the variance explained by each latent component [62,68]. Wavenumbers with VIP > 1.0 were considered significant predictors [85].

Standardized regression coefficients were calculated to assess the direction and magnitude of each spectral region’s unique association with PMI after accounting for inter-correlations among wavenumbers. Standardized coefficients were obtained by multiplying raw PLS coefficients by the standard deviation of each corresponding X-variable, rendering them comparable across spectral regions. Bootstrap resampling (100 iterations) was used to estimate 95% confidence intervals for these coefficients [86]. Positive coefficients indicate bands that increase with PMI; negative coefficients indicate bands that decrease.

Due to non-normal residual distribution (Shapiro–Wilk test, *p* = 0.045), 95% prediction intervals for unknown samples were estimated using non-parametric bootstrap resampling (1000 iterations) of cross-validated residuals [87]. Prediction uncertainty was also reported using percentiles of absolute errors from the final model (50th, 75th, and 90th percentiles).

#### 4.4.5. Binary Classification of Samples

For binary classification of early (≤48 h) versus late (>48 h) PMI, we evaluated several classification methods: Linear Discriminant Analysis (LDA) [88], Quadratic Discriminant Analysis (QDA) [89], Support Vector Machines (SVM) with linear and radial basis function (RBF) kernels [90], and Random Forest [91]. All classifiers were applied to the same preprocessed spectral data used for PLS regression (1800–700 cm^−1^, EMSC normalization and mean-centered second derivatives based on the parameters derived from the 20 samples of known PMI).

Model performance was assessed using LOOCV, with evaluation metrics including accuracy, precision, recall, and F1-score. The confusion matrix was examined to identify systematic misclassification patterns. Following LOOCV evaluation, the best-performing classifier (Random Forest) was retrained on all 20 known samples to serve as the final binary classification model for unknown samples. To identify the spectral features most influential in the Random Forest model, feature importance scores were calculated based on the mean decrease in impurity (Gini importance) across all decision trees [92].

#### 4.4.6. Comparative Analysis of Regression Methods

Due to their distinct principles, ordinary least squares (OLS) [61], partial least squares (PLS) [61], Huber regression [93], Theil–Sen Regression [94] and RANSAC (RANdom SAmple Consensus) [95] were selected to test their capability in predicting PMI based on our spectra. All regression methods were applied to the same preprocessed spectral data and were evaluated using LOOCV.

Performance was assessed on four datasets: (i) all 20 known samples, (ii) excluding clinical confounders (490, 718, 16), (iii) excluding PLS-flagged problematics (490, 718), and (iv) a forensically relevant subset with PMI < 72 h (n = 13). Model performance was evaluated using RMSE, MAE, and R^2^.

## 5. Conclusions

This study successfully developed a practical FTIR-based method for PMI estimation using human VH, with direct applicability to forensic casework. The two-component PLS model demonstrated robust performance across heterogeneous samples, achieving a cross-validated RMSE of 15.82 h on the full dataset and providing credible predictions for ten independent unknown samples with only estimated PMI. The model’s ability to maintain stable performance (RMSE = 12.44 h, R^2^ = 0.705) in the presence of clinically complex cases which caused failure in OLS and RANSAC supports its suitability for real-world forensic applications where case histories are inevitably diverse and unpredictable.

The integrated analytical approach, combining univariate correlation, PCA, PLS regression with standardized coefficients, VIP scores, and binary classification, identified glycoprotein degradation (1190–1192 cm^−1^) and phospholipid membrane breakdown (734–739 cm^−1^) as the dominant PMI markers, refining the set of diagnostically useful spectral features beyond traditional protein and carbohydrate bands. The Random Forest binary classification model provides a practical triage tool with 70% accuracy, outperforming LDA (60%), and confidence scores exceeding 0.9 for 70% of unknown samples, enabling forensic practitioners to prioritize reliable predictions. The observation that prolonged refrigerated storage (sample 641) decouples biochemical from chronological age suggests the necessity for developing temperature-corrected models, which will further enhance forensic utility.

In conclusion, this exploratory study demonstrates the potential of FTIR-based PMI estimation to move toward practical forensic applications by validating model performance on truly unknown samples, establishing PLS as uniquely robust to clinical heterogeneity, providing confidence-scored binary classification (Random Forest) for rapid triage, and identifying glycoprotein degradation and phospholipid membrane breakdown as superior, stable biomarkers. The method is rapid, requires minimal sample preparation, and can be implemented with standard FTIR instrumentation, suggesting its potential transferability to forensic laboratories. However, given the limited sample size, these findings should be considered preliminary. Future studies with larger, more diverse datasets spanning broader PMI ranges and incorporating temperature histories will enable further refinement and eventual integration into routine forensic casework.

## Figures and Tables

**Table 1:** The diagnostic peaks in VH FTIR spectra analyzed here relative to PMI. Literature values [44], observed wavenumbers, vibrational modes, and biochemical assignments [42,44,49] are provided.

Wavenumber of Diagnostic Peak (cm^−1^)		Vibrational Mode	Biochemical Band Assignment
Literature	Observed		
~1666	~1663	ν (C=O), ν (C–N) (amide I)	α helices in peptides, proteins
~1630	~1630	ν (C=O), ν (C–N) (amide I)	β sheets in peptides, proteins; contribution from small nitrogenous solutes
~1580	~1580	ν (C–N), δ (N–H) (amide II)	proteins and nitrogen-containing metabolites (urea, creatinine, uric acid)
~1452	~1456	δ (CH_2_), δ (CH_3_)	lipids, proteins, aliphatic groups from small metabolites
~1417	~1414	ν_s (COO^−^)	free amino acids, organic acids, hyaluronic acid
~1313	~1315	ν (C–N), δ (N–H) (amide III)	proteins, peptides
~1120	~1121	ν (C–O)	lactate and small carbohydrates
~1080	~1086	ν (P–O), ν (C–O)	phosphate-containing compounds, phosphoric acids
~1040	~1041	ν (C–O), ν (C–OH)	glucose, monosaccharides
~925	~925	ν (C–O), ν (P–O)	nucleic acids fragments
~854	~855	ν (C–C), ν (C–O)	lactate and small carbohydrates
~780	~780	ν (C–O), ν (C–C)	carbohydrate

**Table 2:** Correlation of diagnostic peak intensities with PMI expressed as Pearson correlation coefficients (r) with 95% confidence intervals. *p*-values are corrected for multiple testing using the Benjamini–Hochberg false discovery rate (FDR) method.

Peak (cm^−1^)	r	95% CI	p (FDR)	Interpretation
1086	0.549	[0.375, 0.686]	1.6 × 10^−6^	Strong positive
1580	−0.493	[−0.643, −0.307]	2.0 × 10^−5^	Moderate negative
1315	−0.481	[−0.634, −0.292]	2.6 × 10^−5^	Moderate negative
1630	0.434	[0.237, 0.597]	1.7 × 10^−4^	Moderate positive
1041	0.407	[0.205, 0.575]	4.3 × 10^−4^	Moderate positive
1663	0.33	[0.119, 0.513]	5.6 × 10^−3^	Weak positive
1456	0.323	[0.111, 0.506]	6.0 × 10^−3^	Weak positive
1414	−0.29	[−0.479, −0.075]	1.2 × 10^−2^	Weak negative
780	−0.29	[−0.479, −0.075]	1.2 × 10^−2^	Weak negative
855	−0.266	[−0.459, −0.049]	2.1 × 10^−2^	Weak negative
1121	−0.142	[−0.351, 0.080]	0.226	Not significant
925	0.023	[−0.197, 0.242]	0.837	Not significant

**Table 3:** Performance metrics * of the final PLS regression model (2 components) for PMI estimation.

Metric	Value
Samples used	20 (all known)
Optimal components	2
RMSE (hours)	15.82
MAE (hours)	12.27
R^2^	0.531
95% CI (non-parametric)	±36.1

* Metrics are based on LOOCV on all 20 known samples. Non-parametric 95% confidence intervals were calculated by bootstrap resampling of cross-validated residuals (1000 iterations). This approach was necessary to calculate confidence intervals because the residuals have constant variance (homoscedastic) according to Levene’s test (*p* = 0.440), but deviate from normality according to Shapiro–Wilk test (*p* = 0.047).

**Table 4:** Predicted PMI values for unknown samples based on the final PLS model (2 components, calibrated on all 20 known samples). Prediction uncertainty is reported using percentiles of absolute errors from the final model (50th percentile = 7 h; 75th percentile = 16 h; 90th percentile = 20 h). The 95% confidence interval (±36.1 h) was estimated using non-parametric bootstrap resampling of cross-validated residuals (1000 iterations).

Sample	Estimated PMI (h)	PLS Predicted (h)	50% CI (h)	75% CI (h)	90% CI (h)	Agreement with Estimate
647	~12	29.6	[23–37]	[14–46]	[10–50]	Consistent (within 90% CI)
205	~15	67	[60–74]	[51–83]	[47–87]	Discrepant (outside 90% CI)
634	~17	37.7	[31–45]	[22–54]	[18–58]	Discrepant (outside 90% CI)
723	~19	28.9	[22–36]	[13–45]	[9–49]	Consistent (within 75% CI)
643	~20	54.6	[48–62]	[39–71]	[35–75]	Discrepant (outside 90% CI)
637	~21	48.5	[42–56]	[33–65]	[29–69]	Discrepant (outside 90% CI)
467	~33	27.1	[20–34]	[11–43]	[7–47]	Consistent (within 50% CI)
1247	~33	39.6	[33–47]	[24–56]	[20–60]	Consistent (within 50% CI)
598	~35.5	39.2	[32–46]	[23–55]	[19–59]	Consistent (within 50% CI)
641	~179	80.1	[73–87]	[64–96]	[60–100]	Large discrepancy

**Table 5:** Performance comparison of binary classification methods for early (≤48 h) vs. late (>48 h) PMI. Metrics are based on LOOCV.

Classifier	Accuracy	Precision (Early)	Recall (Early)	Precision (Late)	Recall (Late)
Random Forest	0.700	0.750	0.375	0.688	0.917
LDA	0.600	0.500	0.375	0.643	0.750
SVM (linear)	0.600	0	0	0.600	1.000
QDA	0.450	0.412	0.875	0.667	0.167
SVM (RBF)	0.450	0	0	0.529	0.750

**Table 6:** Random Forest-based binary classification results for unknown samples. The model distinguishes early (≤48 h) from late (>48 h) PMI. Confidence scores represent the proportion of decision trees voting for the predicted class.

Sample	Predicted Class	Confidence	PMI Estimate	Agreement
647	Early (≤48 h)	0.59	~12 h	yes
205	Early (≤48 h)	0.52	~15 h	yes
634	Early (≤48 h)	0.64	~17 h	yes
723	Early (≤48 h)	0.83	~19 h	yes
643	Late (>48 h)	0.52	~20 h	no
637	Early (≤48 h)	0.64	~21 h	yes
1247	Early (≤48 h)	0.64	~33 h	yes
467	Early (≤48 h)	0.62	~33 h	yes
598	Early (≤48 h)	0.64	~35.5 h	yes
641	Late (>48 h)	0.87	~179 h	no

**Table 7:** Performance comparison of regression methods under different sample selections. Metrics are based on leave-one-out cross-validation.

Model	Full Dataset (n = 20)			Excluding Clinical Confounders ^i^ (n = 17)			Excluding PLS Problematic ^ii^ (n = 18)			Forensic Range (PMI < 72 h, n = 13)		
	RMSE (h)	R^2^	MAE (h)	RMSE (h)	R^2^	MAE (h)	RMSE (h)	R^2^	MAE (h)	RMSE (h)	R^2^	MAE (h)
PLS (2 comp)	15.82	0.531	12.27	12.27	0.688	9.96	12.44	0.705	10.01	20.46	−2.102	14.90
OLS (Baseline)	15.57	0.545	12.66	10.93	0.753	9.17	18.17	0.370	13.07	20.46	−2.104	14.02
Theil–Sen	15.57	0.545	12.66	10.93	0.753	9.17	18.17	0.370	13.07	20.46	−2.104	14.02
RANSAC	15.91	0.525	13.22	10.93	0.753	9.17	18.19	0.369	13.10	20.75	−2.192	15.11
Huber	24.33	−0.11	21.04	23.08	−0.102	19.99	23.94	−0.093	21.22	13.04	−0.260	10.73

^i^ Excluding samples with documented clinical confounders: 490 (thermal burn), 718 (head trauma), 16 (cancer/stroke). ^ii^ Excluding samples flagged as problematic by the 2-component PLS model: 490, 718 (keeps sample 16).

**Table 8:** Selected samples of known and unknown PMI.

Sample	Case	PMI	Age	Gender	Cause of Death
Cohort 1, hospital deaths, known PMI					
172	172/2025	24.8 *	73	F	thermal burns
490	490/2024	26.67 *	67	M	thermal burns
371	371/2024	29.25 *	72	M	poisoning
152	152/2025	31.83 *	78	M	traumatic brain injury, chronic subdural hematoma
484	484/2025	40.5 *	79	M	traumatic brain injury
1148	1148/2024	43.58 *	46	M	traumatic brain injury
1400	1400/2024	43.83 *	51	M	traumatic brain injury
718	718/2024	46.25 *	50	M	head trauma, subdural hematoma surgery
1242	1242/2024	48.73 *	57	M	epilepsy seizure
455	455/2025	48.97 *	49	M	tonsil carcinoma
645	645/2024	53.42 *	65	M	traumatic brain injury
1139	1139/2024	55.98 *	71	M	cervical spine injury
1285	1285/2024	64.87 *	90	F	septic shock, infected leg hematoma
317	317/2024	73.33 *	75	M	subdural hematoma, septic shock
482	482/2025	76.08 *	71	M	septic shock
1478	1478/2024	82.83 *	64	M	traumatic brain injury
597	597/2024	87.25 *	88	M	traumatic brain injury
332	332/2024	90.5 *	48	M	meningeal hemorrhage
16	16/2025	94.33 *	57	M	pancreatic cancer, acute cerebral stroke
1104	1104/2024	97.58 *	54	F	drug intoxication
Cohort 2, estimated PMI					
Hospital deaths					
641	641/2024	179.28 *	69	M	septic shock
Scene deaths					
647	647/2024	~12 **	35	M	drug intoxication
205	205/2025	~15 **	55	M	hypothermia
634	634/2024	~17 **	64	M	fall from height
723	723/2024	~19 **	19	M	hypothermia
643	643/2024	~20 **	55	M	fall from height
637	637/2024	~21 **	71	F	thermal burns
467	467/2025	~33 **	49	M	choking
1247	1247/2024	~33 **	55	M	septic shock
598	598/2024	~35.5 **	25	M	hanging

* known PMI was calculated as the difference between date and time of autopsy (and the subsequent sample collection) and date and time of death in the hospital (Equation (S2), Supplementary Material S3). ** PMI was estimated as the difference between the date and time of autopsy (and subsequent sample collection) and date and time of body recovery at the scene (Equation (S3), Supplementary Material S3).

## Data Availability

The raw data supporting the conclusions of this article will be made available by the authors on request.

## Supplementary Materials

The following supporting information can be downloaded at: https://www.mdpi.com/article/10.3390/ijms27083468/s1.

## Abbreviations

The following abbreviations are used in this manuscript:

PMI	postmortem interval
VH	vitreous humor
ATR	attenuated total reflection
FTIR	Fourier-transform infrared spectroscopy
PCA	principal component analysis
PC	principal components
PLS	partial least squares
LOOCV	leave-one-out cross-validation
RMSE	root mean squared error
MAE	mean absolute error
VIP	variable importance in projection
LDA	linear discriminant analysis
OLS	ordinary least squares
RE	right eye
LE	left eye
EMSC	extended multiplicative signal correction
R^2^	coefficient of determination
RANSAC	RANdom SAmple Consensus
