# Supplementary material for: FTIR Spectroscopy of Vitreous Humor for Postmortem Interval Estimation: A Multivariate Regression Approach
Source: Int J Mol Sci. 2026 Apr 13;27(8):3468. doi: 10.3390/ijms27083468 (PMC13115575; doi:10.3390/ijms27083468)
Supplement: Supplementary file 1 [file ijms-27-03468-s001.zip › ijms-4214800-supplementary.pdf]

FTIR spectroscopy of vitreous humor for post-mortem interval estimation: A multivariate regression approach

Ioana Ruxandra Țurlea [1,2], George Cristian Curcă [1,2], Maria Mernea [3,*], Alina Cristina Mătanie [3], Sergiu Fendrihan [4] and Dan Florin Mihăilescu [3]

[1] Department of Legal Medicine and Bioethics, "Carol Davila" University of Medicine and Pharmacy, 8 Eroii Sanitari Boulevard, 050474 Bucharest, Romania; ioana-ruxandra.turlea@umfcd.ro (I.R.Ț.); george.curca@umfcd.ro (G.C.C.);

[2] "Mina Minovici" National Institute of Legal Medicine, 9-11 Vitan-Bârzești Road, 077160 Bucharest, Romania;

[3] Department of Anatomy, Animal physiology and Biophysics, Faculty of Biology, University of Bucharest, 91–95 Splaiul Independenței Str., 050095 Bucharest, Romania; maria.mernea@bio.unibuc.ro (M.M.), cristina.matanie@bio.unibuc.ro (A.C.M.); d.f.mihailescu@gmail.com (D.F.M.);

[4] Non-Governmental Research Organization Biologic, 14 Schitului Str., 032044 Bucharest, Romania; ecologos23@yahoo.com (S.F.)

* Correspondence: maria.mernea@bio.unibuc.ro

Supplementary material 1. Hierarchical clustering of FTIR spectra based on their root mean squared deviation.

To explore the clustering of VH samples based on their FTIR spectral profiles, we applied an unsupervised hierarchical clustering analysis. We calculated pairwise distances between spectra using the root mean square deviation (RMSD) metric, according to Eq. (1):

$$RMSD\ (a,b) = \sqrt{\frac{1}{n}(a_i - b_i)^2} \qquad \text{(Eq. 1)}$$

where a and b are the two spectra and n is the number of wavelengths in the 1800 – 700 cm^{-1} range.

Based on the on the pairwise RMSD matrix considering all possible spectra pairs we performed an agglomerative hierarchical clustering using the unweighted pair group method with arithmetic mean (UPGMA, average linkage). Cluster validity was assessed using multiple complementary approaches: cophenetic correlation coefficient, silhouette analysis, bootstrap resampling (100 iterations) and inconsistency coefficients. All analyses were performed in Python (v3.11.7) using SciPy and scikit-learn libraries, with dendrograms visualized using Matplotlib.

The resulting hierarchical clustering of the 30 FTIR spectra (20 known PMI, 10 unknown PMI) is showed in Figure S1. The dendrogram reveals a clear separation into two main clusters. The cophenetic correlation coefficient of 0.9118 indicated a good preservation of the original pairwise distance matrix in the dendrogram structure. Silhouette analysis identified two clusters as optimal, with a mean silhouette width of 0.6379. This value indicates well-separated clusters with good internal cohesion.

Bootstrap resampling demonstrated low cluster stability (Adjusted Rand Index = 0.0181 ± 0.1546), indicating that the exact assignment of individual samples to clusters is sensitive to sampling variations, which can be explained by the small dataset analyzed. This shows that the two-cluster structure is statistically discernible, but the precise cluster membership of individual samples should be interpreted with caution. The inconsistency coefficients, which measure the relative dissimilarity of merged clusters, showed low values for early merges (indicating tight, natural clusters) and higher values (0.707–0.887) for

merges 5–7, corresponding to the separation of the two main clusters. This pattern confirms that the two-cluster structure represents a genuine hierarchical division in the data.

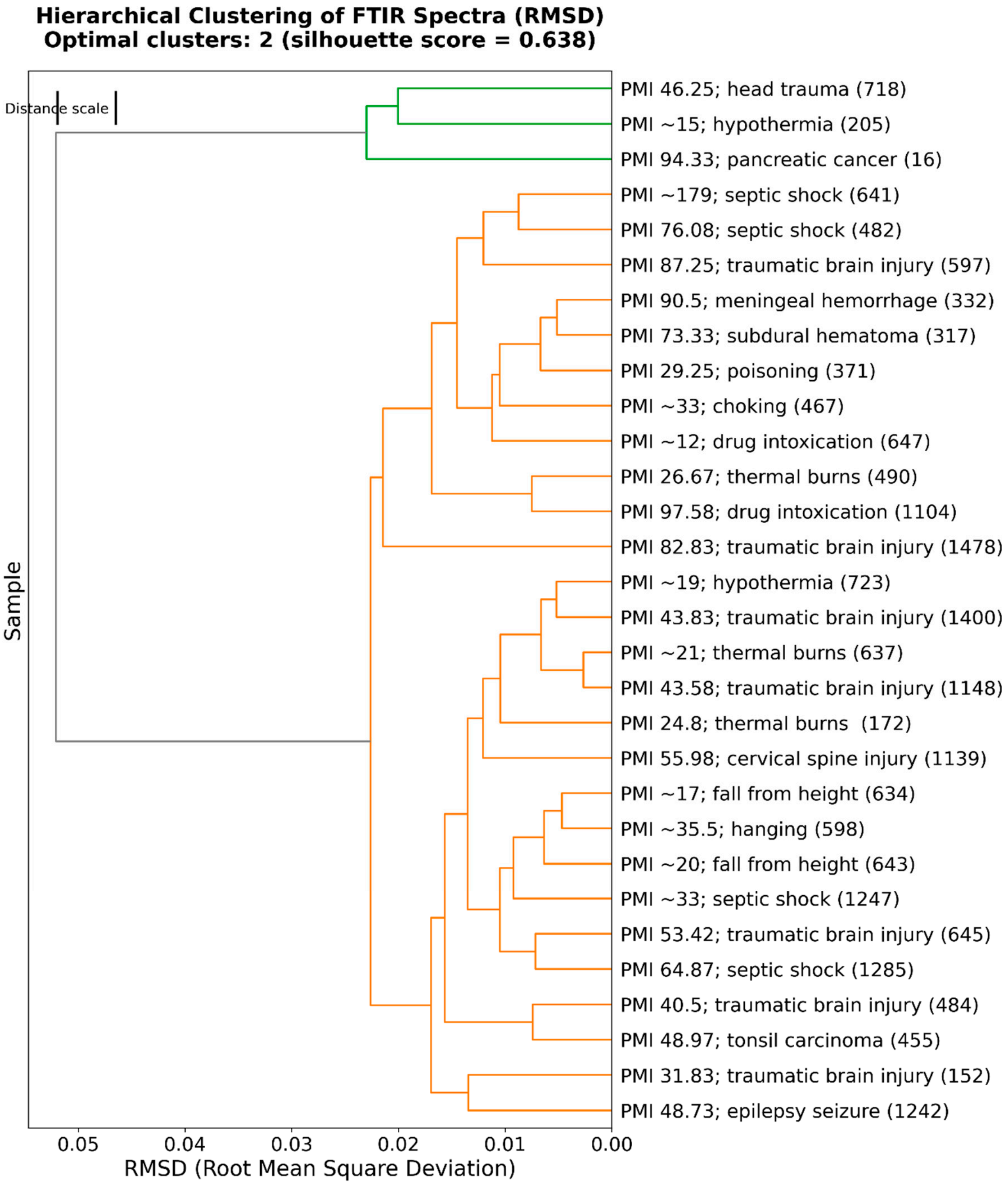

Figure S1.1. Horizontal dendrogram showing the relationship among all 30 vitreous humor samples. Labels indicate PMI, cause of death, and sample ID. The optimal two-cluster solution (silhouette score = 0.636) is indicated by color shading: Cluster 1 (blue, n=27) and Cluster 2 (orange, n=3).

As can be seen in the dendrogram (Figure S1), Cluster 1 contained the majority of samples (27/30), encompassing a wide range of PMI values (24.8–97.6 h) and diverse causes of death including thermal burns, traumatic brain injury, septic shock, and drug intoxication.

Cluster 1 comprises sample 490, the outlier revealed by the analysis of absorption peaks intensities for 20 – 30 hours PMI range. Overall, the cluster comprises other thermal burns cases, including case 172 that has a close PMI of 24.8 h. In the case 172 we identified a different casuistry, with obesity, hyperuricemia, dyslipidemia, heart failure and only 9 days hospitalization. This shows that spectra are sensitive to the particular biochemical conditions of each sample, even if the cause of death is similar.

Cluster 2 comprised only three samples: 16 (PMI 94.3 h, pancreatic cancer, acute cerebral stroke), 205 (PMI ~15 h, hypothermia), and 718 (PMI 46.2 h, head trauma and subdural hematoma surgery). This shows that, despite their different PMI and primary cause of death, the three samples exhibit spectral similarities that likely arise from convergent biochemical pathways activated by acute physiological stress and blood-ocular barrier disruption. In samples 16 and 718, intracranial pathology (stroke and head trauma, respectively) promotes breakdown of the blood-ocular barrier, allowing blood-derived proteins, inflammatory mediators, and cellular debris to enter the vitreous humor [1–3].

For sample 205 (hypothermia), a different mechanism may produce a similar spectral outcome. The stress induced by hyperthermia was also showed to impact the composition of VH by elevating the levels of glucose [4] along with 28 metabolites (21 phosphatidylcholines, 3 sphingomyelins, spermine, citrulline, acetylcarnitine, and hydroxybutyrylcarnitine) [5], while decreasing the levels of 5 other metabolites (methionine sulfoxide, tryptophan, phenylalanine, alanine, and ornithine) [5]. These metabolite shifts affect the same spectral regions influenced by blood-ocular barrier disruption, particularly phosphate-containing compounds (~1083 cm^{-1}), carbohydrates (~1041 cm^{-1}), and protein-related bands (~1663, ~1630 cm^{-1}). These could explain the convergent spectral features observed across Cluster 2.

Still, it is important to stress that the above interpretation remains tentative. These hypotheses are offered as plausible explanations based on the existing literature, but we have not conducted additional biochemical or metabolomic analyses to directly confirm them. Moreover, the association between spectral shape and specific causes of death was not a primary purpose of this study.

Overall, the spectra clustering results showed that the grouping is insensitive to PMI or cause of death, which suggests that other factors might contribute to the biochemical variability of samples, like the comorbidities, agonal states and therapeutic interventions prior to death. The three samples forming a separate cluster (16, 205, 718) represent exceptions that may reflect particularly pronounced physiological stress, as discussed above. A methodological limitation of the clustering analysis can be the small number of spectra that were investigated, which may affect the generalizability of the cluster assignments.

Supplementary material 2. PLS model validation and sample reliability

Model Validation Using Multiple Train/Test Splits

To validate the PLS model, we performed five iterations in which spectra of known PMI were split randomly into the training set (15 samples) and test set (5 samples). The results are summarized in Table S2.1. Across the 5 splits, the mean test RMSE was 17.9 ± 7.1 h (range 6.7–28.8 h), reflecting the heterogeneity of the dataset.

Test set performance showed considerable variability (RMSE range: 6.7–28.8 h, R^2 range: -0.33 to 0.89), reflecting the heterogeneity of the dataset. Splits that included clinically complex samples (16, 490, 718) in the test set yielded the poorest performance (Split 4: RMSE = 28.75 h, R^2 = -0.33; Split 5: RMSE = 19.64 h, R^2 = 0.38), while splits containing more homogeneous samples achieved excellent performance (Split 2: RMSE = 6.70 h, R^2 = 0.89). These results confirm that the model's performance is strongly influenced by the inclusion of challenging cases, and that the 2-component model (most frequent choice) provides a parsimonious and robust solution.

Table S2.1. PLS model performance across 5 random train/test splits. Preprocessing (EMSC, second derivative, mean-centering) was performed using training set parameters only to prevent data leakage.

**Split**	**Test Samples**	**Optimal Components**	**Test RMSE (h)**	**Test R^2**	**Test MAE (h)**
1	172, 332, 1478, 490, 1242	2	18.9	0.529	15.71
2	482, 1148, 484, 332, 1242	4	6.7	0.886	5.44
3	455, 317, 172, 597, 371	10	15.43	0.598	13.27
4	16, 371, 1478, 718, 1148	3	28.75	-0.332	21.52
5	597, 1139, 1478, 490, 16	2	19.64	0.383	12.77
Mean ± SD	—	—	17.9 ± 7.1	0.41 ± 0.41	13.7 ± 5.2

Sample Reliability and Error Analysis based on the final PLS model.

Beyond aggregate performance metrics, examining prediction errors at the individual sample level provides valuable insight into model robustness and identifies cases that may be influenced by confounding factors. Based on the absolute prediction errors from leave-one-out cross-validation (LOOCV) on all 20 known samples using the final 2-component PLS model (RMSE = 15.8 h), samples were categorized into three reliability groups (Table S2.2):

- Reliable samples (error < RMSE, n = 14): Predictions within 15.8 h of true PMI
- Borderline samples (RMSE ≤ error < 2×RMSE, n = 4): Moderate deviations (15.8 – 31.6 h)
- Problematic samples (error ≥ 2×RMSE, n = 2): Substantial deviations requiring scrutiny.

Table S2.2. Classification of known samples by prediction error magnitude for the final PLS model.

Reliability Group	Threshold	Number of Samples	Examples (Sample ID, True PMI, Predicted PMI, Error)
**Reliable**	error < 15.8 h	14	**482**: 76.08→75.98 h (0.1 h); **172**: 24.8→23.83 h (1.0 h); **1242**: 48.73→50.02 h (1.3 h); **1148**: 43.58→45.42 h (1.8 h); **645**: 53.42→55.28 h (1.9 h); **16**: 94.33→98.64 h (4.3 h)
**Borderline**	15.8 – 31.6 h	4	**1478**: 82.83→65.21 h (17.6 h); **332**: 90.5→71.77 h (18.7 h); **597**: 87.25→67.83 h (19.4 h); **371**: 29.25→49.86 h (20.6 h)
**Problematic**	≥ 31.6 h	2	**490:** 26.67→62.80 h (36.1 h); **718:** 46.25→79.52 h (33.3 h)

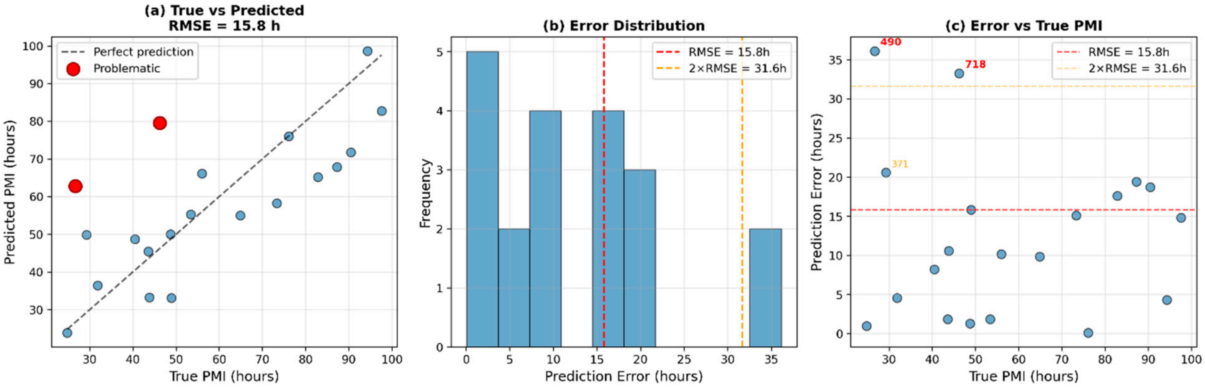

Figure S2.1. Detailed performance assessment of the PLS regression model based on leave-one-out cross-validation (LOOCV). (a) Predicted versus true PMI values for all 20 known samples. The black dashed line represents perfect prediction (y = x). The model achieves a cross-validated RMSE of 15.8 h. The samples labelled in red (490 and 718) were identified as problematic (error > 2×RMSE). (b) Distribution of absolute prediction errors. The red and orange dashed lines indicate the RMSE (15.8 h) and twice the RMSE (31.6 h), respectively. Samples with errors below the RMSE (n=14) are considered reliable, while those above twice the RMSE (n=2) are flagged as problematic. (c) Prediction error plotted against the true PMI value. Annotated samples have errors exceeding 31.6 h.

The two problematic samples were 490 (true PMI: 26.7 h, predicted: 62.3 h, error: 36.1 h) and 718 (true PMI: 46.2 h, predicted: 79.5 h, error: 33.3 h). Both samples correspond to cases with documented clinical confounders: sample 490 (thermal burn) and sample 718 (head trauma with surgery). Their identification as problematic is consistent with their atypical spectral profiles observed in PCA and their substantial overestimation in the independent test set (sample 490).

In contrast, highly reliable predictions were obtained for samples spanning the entire PMI range, including:

- Early PMI: sample 172 (24.8 h true, 23.8 h predicted, error 1.0 h);
- Mid-range: sample 1242 (48.7 h true, 50.0 h predicted, error 1.3 h); sample 645 (53.4 h true, 55.3 h predicted, error 1.9 h);
- Late PMI: sample 482 (76.1 h true, 76.0 h predicted, error 0.1 h); sample 16 (94.3 h true, 98.6 h predicted, error 4.3 h).

Supplementary material 3. Detailed description of analyzed cases.

Cohort 1 – hospital deaths, known PMI

A first cohort, Cohort 1 comprises 20 patients hospitalized that died in the hospital with violent or non-violent causes of death (Table S3.1). Selection criteria: hospitalized cases with known post-mortem interval, PMI known (hospital data: patient file). Exclusion criteria: cases non-hospitalized with post-mortem interval unknown. The known PMI formula applied in these cases is:

Known PMI = (DOA at TOA) – (DOD at TOD) (Eq 2).

Explanations. The Date of Autopsy (DOA) and Time of Autopsy (TOA) are known from the autopsy report. The Date of Death (DOD) and Time of Death (TOD) are documented in the patient file from the hospital. After body recovery within the hospital, the cadavers were kept refrigerated at 4 ^{0}C.
Example. For example, in case no. 172/2025, known **PMI formula** becomes (Date of Autopsy, DOA at time of autopsy, TOA) – (Date of Death, DOD at time of death, TOD) = (13.02.2025 at 08:50) – (12.02.2025 at 08:02) = 24.8 hours.

Table S3.1. Cohort 1, gender age group, date of death (DOD), time of death (TOD), date of autopsy (DOA), time of autopsy (TOA), calculated PMI (PMI) and the cause of death.

No	Case No.	Gender	Age	DOD	TOD	DOA	TOA	PMI hours	Cause of death
1	172/ 2025	F	73	12.02.2025	08:02	13.02.2025	08:50	24.8	Thermal burns
2	490/ 2024	M	67	22.04.2024	05:50	23.04.2024	08:30	26.67	Thermal burns
3	371/ 2024	M	72	25.03.2024	04:35	26.02.2024	09:50	29.25	Poisoning
4	152/ 2025	M	78	06.02.2025	03:00	07.02.2025	10:50	31.83	Traumatic brain injury
5	484/ 2025	M	79	21.04.2025	19:15	23.04.2025	11:45	40.5	Traumatic brain injury
6	1148/ 2024	M	46	24.09.2024	13:10	26.09.2024	08:45	43.58	Traumatic brain injury
7	1400/ 2024	M	51	25.11.2024	13:55	27.11.2024	09:45	43.83	Traumatic brain injury
8	718/ 2024	M	50	16.06.2024	11:25	18.06.2024	09:40	46.25	Head trauma
9	1242/ 2024	M	57	21.10.2024	08:46	23.10.2024	09:30	48.73	Epilepsy seizure
10	455/ 2025	M	49	13.04.2025	09:22	15.04.2025	10:20	48.97	Tonsil carcinoma
11	645/ 2024	M	65	29.05.2024	04:05	31.05.2024	09:30	53.42	Traumatic brain injury
12	1139/ 2024	M	71	23.09.2024	00:46	25.09.2024	08:45	55.98	Cervical spine injury
13	1285/ 2024	F	90	29.10.2024	16:08	01.11.2024	09:00	64.87	Septic shock
14	317/ 2024	M	75	10.03.2024	07:35	13.03.2025	08:55	73.33	Subdural hematoma
15	482/ 2025	M	71	20.04.2025	07:10	23.04.2025	11:15	76.08	Septic shock
16	1478/ 2024	M	64	14.12.20 24	23:40	18.12.2024	10:30	82.83	Traumatic brain injury
17	597/ 2024	M	88	17.05.2024	17:15	21.05.2024	08:30	87.25	Traumatic brain injury

No	Case No.	Gender	Age	DOD	TOD	DOA	TOA	PMI hours	Cause of death
17	332/ 2024	M	48	14.03.2024	14:05	18.03.2025	08:35	90.5	Meningeal hemorrhage
19	16 025	M	57	04.01.2025	11:10	08.01.2025	09:30	94.33	Metastatic pancreatic cancer
20	1104/ 2024	F	54	13.09.2024	08:30	17.09.2024	10:05	98.58	Acute drug intoxication

Cohort 2 – scene deaths, unknown PMI

A second cohort, Cohort 2 includes 10 cases (Table S3.2). Nine cases are violent scene deaths, all involving unidentified persons. These are non-hospital cases, for which the PMI was only estimated. Selection criteria: non-hospitalized cases with post-mortem interval estimated only. Exclusion criteria: hospitalized cases. The estimated PMI formula applied in these cases is:

Estimated PMI = (DOA at TOA) – (DOBR at TOBR)   (Eq. 3).

Explanations. Date of Autopsy (DOA) and Time of Autopsy (TOA) are known from the autopsy report. Date of Body Recovery (DOBR) and Time of Body Recovery (TOBR) are known from the police report. The Date of Body Recovery (DOBR) include date when the police officers arrived at the death scene, the police investigation, and body transportation to the morgue (if an autopsy is ordered). Date of death (DOD) are estimated using postmortem changes (e.g. body decay phases, body temperature). The Time of Death (TOD) usually remains unknown except in rare cases.

Example. For example, in case no. 647/2024, PMI EST formula = (DOA at TOA) – (DOBR at TOBR) become (31.05.2024 at 09:00) – (30.05.2024 at 22:00) = ~12 hours. After recovery from the death scene, the bodies are transported to the morgue, where they are kept refrigerated at 4° C until the autopsy.

In addition to the 9 cases of secene deaths, Cohort 2 included sample 641 that was a hospital death with a PMI of ~179 hours. Although its PMI is known, the sample was not included in Cohort 1 with the following reasons:

1. Temporal discontinuity: The 79 h gap between sample 641 and the next highest PMI (97.6 h) would have required the model to extrapolate across an uncharacterized region, potentially distorting the degradation trajectory estimated from the densely sampled 25–100 h range.

2. Biochemical atypicality: Preliminary PCA revealed that sample 641 clustered with samples of significantly lower PMI (data not shown), consistent with its documented refrigeration history. Including this biochemically decoupled sample in the training set would have introduced a confounding variable not representative of typical post-mortem progression.

3. Forensic design: By assigning sample 641 to the unknown cohort, we could directly test the model's ability to detect temperature-modulated biochemical age, a critical feature for real-world applications where thermal history is often unknown. The resulting prediction (80.5 h vs. 179 h estimated) confirmed that FTIR detects biochemical rather than chronological age, validating this methodological choice.

Table S3.2. Cohort 2, gender, age group, date of body recovery (DOBR), time of body recovery (TOBR), date of death (DOD_{CPM} - estimated with conventional postmortem), date of autopsy, DOA, TOA, estimated PMI (PMI_{Est}; hours), cause of death. DOBR is similar with DOD_{CPM}. Using conventional methods in the 9 scene deaths of Cohort 2, we observed that conventional postmortem changes indicate that the person has died in the same day with DOBR.

No	Case No.	Gender	Age	DOBR	TOBR	DOD_{CPM}	DOA	TOA	PMI_{EST} hours	Cause of death
1	647/ 2024	M	35	30.05.2024	22:00	~30.05.2024	31.05.2024	09:00	~12	Acute drug intoxication
2	205/ 2025	M	55	19.02.2025	17:30	~19.02.2025	20.02.2025	08:35	~15	Hypotherm ia
6	634/ 2024	M	64	27.05.2024	15:00	~27.05.2024	28.05.2024	08:30	~17	Fall from height
3	723/ 2024	M	19	17.06.2024	15:00	~17.06.2024	18.06.2024	09:40	~19	Hypotherm ia
4	643/ 2024	M	55	29.05.2024	13:00	~29.05.2024	30.05.2024	09:15	~20	Fall from height
7	637/ 2024	F	71	28.05.2024	12:00	~28.05.2024	29.05.2024	09:40	~21	Thermal burns (fire)
5	467/ 2025	M	49	16.04.2025	00:00	~16.04.2025	17.04.2025	09:00	~33	Choking
8	1247/ 2024	M	55	23.10.2024	00:05	~23.10.2024	24.10.2024	08:50	~33	Septic shock
9	598/ 2024	M	25	19.05.2024	22:00	~19.05.2024	21.05.2024	09:00	~35.5	Hanging
10	641/ 2024	M	69	22.05.2024	21:50	~22.05.2024	30.05.2024	09:07	~179.28	Septic shock

Limitations of the constructed cohorts

There are two limitations in PMI_{EST} calculation in our study: 1. The time from death until the body announcement (usually made by call) may vary. 2. PMI includes a non-refrigerated period from death until body recovery and transportation to the morgue and a refrigerated period at the morgue until autopsy. Environmental conditions (high or low temperature), investigation duration, non-refrigerated transportation, etc. may impact the degradation rates.

From a forensic perspective, these limitations are not unique to the present study, they affect all PMI estimation techniques. The value in our approach lies in demonstrating that, despite this unavoidable variability, ATR-FTIR spectroscopy combined with PLS regression can extract a coherent biochemical signal correlated with PMI across diverse cases. The model's ability to identify outlier samples (490, 718) and detect temperature effects (641) further underscores its sensitivity to biologically meaningful variations. In this way, our study establishes a realistic benchmark for what ATR-FTIR spectroscopy can offer in real forensic scenarios and positions ATR-FTIR spectroscopy as a complementary tool that can strengthen PMI estimations in medico-legal practice.

References:

1. Hitomi, E.; Simpkins, A.N.; Luby, M.; Latour, L.L.; Leigh, R.J.; Leigh, R. Blood-Ocular Barrier Disruption in Patients with Acute Stroke. *Neurology* **2018**, *90*, e915–e923, doi:10.1212/WNL.0000000000005123.
2. Shaw, H.E.; Landers, M.B. Vitreous Hemorrhage after Intracranial Hemorrhage. *Am. J. Ophthalmol.* **1975**, *80*, 207–213, doi:10.1016/0002-9394(75)90134-8.
3. Jena, S.; Tripathy, K. Vitreous Hemorrhage. In *StatPearls*; StatPearls Publishing: Treasure Island (FL), 2025.
4. Coe, J.I. Hypothermia: Autopsy Findings and Vitreous Glucose. *J. Forensic Sci.* **1984**, *29*, 389–395.
5. Rousseau, G.; Chao de la Barca, J.M.; Rougé-Maillart, C.; Teresiński, G.; Chabrun, F.; Dieu, X.; Drevin, G.; Mirebeau-Prunier, D.; Simard, G.; Reynier, P.; et al. Preliminary Metabolomic Profiling of the Vitreous Humor from Hypothermia Fatalities. *J. Proteome Res.* **2021**, *20*, 2390–2396, doi:10.1021/acs.jproteome.0c00901.
